# Transcriptional profiling of root-knot nematode induced feeding sites in cowpea (*Vigna unguiculata *L. Walp.) using a soybean genome array

**DOI:** 10.1186/1471-2164-11-480

**Published:** 2010-08-19

**Authors:** Sayan Das, Jeffrey D Ehlers, Timothy J Close, Philip A Roberts

**Affiliations:** 1 Department of Botany and Plant Sciences, University of California, Riverside, CA 92521, USA; 2 Department of Nematology, University of California, Riverside, CA 92521, USA

## Abstract

**Background:**

The locus *Rk *confers resistance against several species of root-knot nematodes (*Meloidogyne spp*., RKN) in cowpea (*Vigna unguiculata*). Based on histological and reactive oxygen species (ROS) profiles, *Rk *confers a delayed but strong resistance mechanism without a hypersensitive reaction-mediated cell death process, which allows nematode development but blocks reproduction.

**Results:**

Responses to *M. incognita *infection in roots of resistant genotype CB46 and a susceptible near-isogenic line (null-*Rk*) were investigated using a soybean Affymetrix GeneChip expression array at 3 and 9 days post-inoculation (dpi). At 9 dpi 552 genes were differentially expressed in incompatible interactions (infected resistant tissue compared with non-infected resistant tissue) and 1,060 genes were differentially expressed in compatible interactions (infected susceptible tissue compared with non-infected susceptible tissue). At 3 dpi the differentially expressed genes were 746 for the incompatible and 623 for the compatible interactions. When expression between infected resistant and susceptible genotypes was compared, 638 and 197 genes were differentially expressed at 9 and 3 dpi, respectively.

**Conclusions:**

In comparing the differentially expressed genes in response to nematode infection, a greater number and proportion of genes were down-regulated in the resistant than in the susceptible genotype, whereas more genes were up-regulated in the susceptible than in the resistant genotype. Gene ontology based functional categorization revealed that the typical defense response was partially suppressed in resistant roots, even at 9 dpi, allowing nematode juvenile development. Differences in ROS concentrations, induction of toxins and other defense related genes seem to play a role in this unique resistance mechanism.

## Background

Cowpea (*Vigna unguiculata *L. Walp) is grown extensively as a food and fodder crop in West Africa, lower elevation areas of eastern and southern Africa, north-eastern Brazil, parts of the Middle East, India, and the south-eastern and south-western regions of North America [1]. In West Africa cowpea is mainly cultivated as a rainfed crop from April to November depending on the location. Cowpea (2N = 2X = 22) has a genome size of ~600 Mbp [2].

Root-knot nematodes (RKN, *Meloidogyne *spp.) are sedentary endoparasites with a wide host range and one of the world's most damaging crop pests [3]. RKN feeding in plant roots leads to development of specialized feeding structures in the vascular parenchyma called "giant cells". The infective stage of this nematode is the second stage-juvenile (J2). J2 penetrate the roots and go through three successive molts to become adult females. Some economically important RKN species, including *M. incognita*, reproduce by obligate mitotic parthenogenesis, while many other RKN species reproduce sexually [4].

In cowpea RKN is an important pest worldwide and host plant resistance has been a preferred strategy along with cultural practices to control the nematode population in infested cowpea fields [5,6]. The *Rk *locus in cowpea has been used extensively to breed root-knot nematode resistant varieties in the USA and other countries. This locus was first designated as *Rk *by Fery and Dukes [7] and it confers resistance to many populations of *M. incognita, M. arenaria, M. hapla *and *M. javanica*.

The *Rk*-mediated resistance in cowpea has been characterized histologically by Das *et al*. [8]. An important finding from that study was that the resistance response was much delayed during the incompatible interaction and there was an absence of typical hypersensitive reaction (HR) mediated cell death in the resistant roots upon nematode infection. This is in contrast to several other plant-RKN systems studied so far which do have a typical HR. For example, *Mi-1 *mediated resistance in tomato triggers a rapid HR as early as 24 hours post-infection [9], and both *Me3*-mediated resistance in pepper [10] and incompatible interactions in soybean [11] show strong early HR.

Whole genome microarrays provide a means to scan for genes involved in particular biological processes on a global scale. Unfortunately cowpea does not yet have a commercially available microarray platform. It was shown previously that the commercially available soybean GeneChip from Affymetrix can be used effectively in cowpea to identify single feature polymorphisms (SFPs) [12]. In that study, cowpea RNA was used as a surrogate for DNA to identify SFPs, which established the utility of the soybean genome array as a satisfactory platform for use in examining cowpea transcripts. In the current study the same soybean platform was used to study the global resistant and susceptible cowpea responses to nematode infection of roots.

There have been several microarray studies of the nematode infection process in plants in last few years. The RKN-plant compatible interactions have been studied using microarrays by several groups in *Arabidopsis *[13,14] and tomato [15,16]. Global gene expression levels also have been studied during the infection process of another important plant parasitic nematode, soybean cyst nematode (*Heterodera glycines*) [17-22]. However, few studies have examined incompatible plant-nematode interactions in resistant plants [16,23,24].

In this study the transcriptome profile of both incompatible and compatible cowpea-RKN interactions for two time points was investigated using the Affymetrix soybean GeneChip. This is the first study of this kind in the cowpea-RKN interaction. It provides a broad insight into the *Rk*-mediated resistance in cowpea and creates an excellent dataset of potential candidate genes involved in both nematode resistance and parasitism, which can be tested further for their role in this biological process using functional genomics approaches.

## Results

### Heterologous microarray platform

In order to elucidate the plant response to root-knot nematodes, infected resistant CB46 (incompatible interaction) was compared with non-infected CB46, and infected susceptible null-*Rk *(compatible interaction) was compared with non-infected null-*Rk*. Two time points were chosen for this analysis i.e., 3 and 9 dpi. Nine dpi was selected as a critical time point because sequentially assayed histological sections during 21 days of infection revealed that at 9 dpi the first subtle differences appeared between incompatible and compatible interactions [8]. The 3-dpi samples provided a time point prior to visible differences histologically, between incompatible and compatible interactions.

The average number of soybean probe sets which had "present" call in 9-dpi samples was 10,521, which comprised 28% of the total number of soybean probe sets on the soybean GeneChip. Similarly, the average number of "present" calls in 3-dpi samples was 10,685 (~28.5% of all soybean probe sets). When soybean RNA was used to hybridize the soybean GeneChip, the "present" call percentage ranged from 70-75% [25]. Therefore about 40% (28/70) of the content of the soybean GeneChip is informative for cowpea.

### Data quality

**Figure 1:**
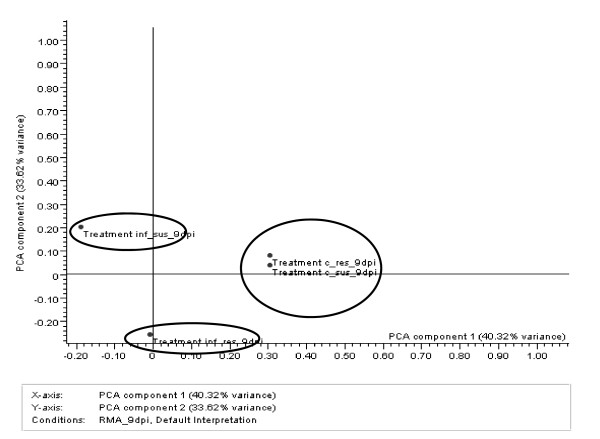
**Principal component analysis (PCA) plot of cowpea genome response to nematode infection**. Each dot represents the mean of a particular condition (treatment).

Principal component analysis (PCA) was done on triplicate data for each treatment at 9 dpi in order to visualize the overall genome response to nematode infection in resistant and susceptible cowpea genotypes. PCA on conditions (treatments) for the 9-dpi sample are shown in Fig. 1. PCA component 1 (40.32% of total variance) and PCA component 2 (33.62% of total variance) comprised the majority of the described variance (73.94%). The PCA showed a clear separation between the two genotypes when infected with nematodes whereas the two genotypes clustered together when there was no external stimulus (non-infected control). Because we used cowpea near-isogenic lines for this analysis, clustering of the non-infected control samples was expected. This confirmed the robustness of the experimental design. The PCA plot for 3-dpi samples is presented in additional file 1.

**Figure 2:**
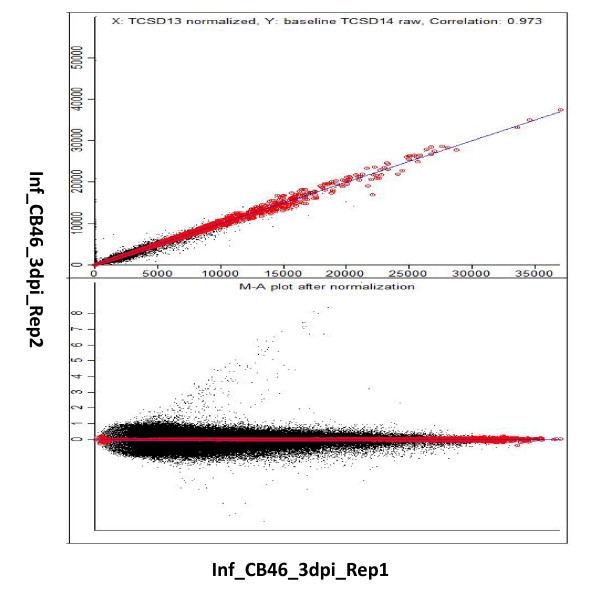
**MA-scatter plot of all probe sets of two replicates of nematode infected resistant CB46 treatment at 3 dpi**. The correlation was calculated on normalized expression values. Top panel represents the invariant probe sets to calculate the median. In the bottom panel all the probe sets were plotted around the median line.

Because there were only two biological replicates for the 3-dpi samples, a correlation analysis was performed between the two replicates of each treatment. A representative MA-scatter plot is shown in Fig. 2. Correlation coefficients between replicates ranged from 0.932 to 0.973 at a p value cut-off of 0.05, showing the robustness of the data (correlation coefficients greater than 0.9 are considered to be high [25]).

### Gene expression in incompatible and compatible interactions in 9-dpi root samples

At 9 dpi three different comparisons were made. In the first comparison gene expression in the resistant CB46 roots infected with root-knot nematodes (incompatible interaction) was compared with non-infected CB46. Secondly, a comparison in gene expression was made between the infected null-*Rk *(compatible interaction) and non-infected null-*Rk*. Finally, a comparison of gene expression was made between the infected *Rk *and infected null-*Rk *near-isogenic lines. The final comparison was important because the near-isogenic lines were predicted to show differential gene expression for the genes which are critical for nematode resistance or nematode parasitism.

**Figure 3:**
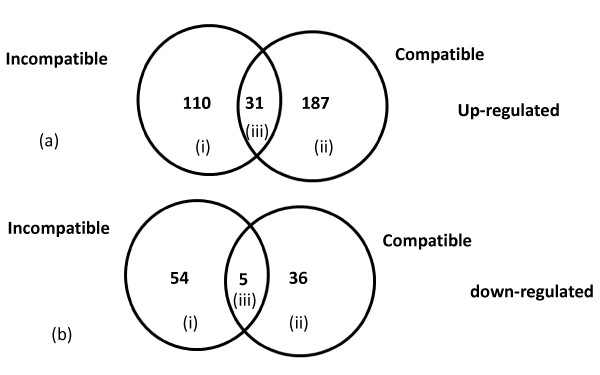
**Venn diagram of differentially expressed genes in incompatible (resistant CB46) and compatible (susceptible null-*Rk*) interactions at 9 dpi**. (a) Genes 1.5-fold or more up-regulated in incompatible (i) and compatible (ii) interactions and genes overlapping between both interactions (iii). (b) Genes 1.5-fold or more down-regulated in incompatible (i) and compatible (ii) interactions and genes overlapping between both interactions (iii).

In the incompatible interaction 552 (~5.3% of total expressed probe sets) genes were significantly differentially expressed between the *Rk*-infected and non-infected treatments based on the statistical test (see materials and methods). These genes were then passed through a 1.5 fold-change filter. The geometric mean of the normalized expression intensities of all samples under one condition were used to calculate the fold-change ratios. If the ratio of geometric means of the infected sample and the non-infected control for a probe set was ≥ 1.5, then that particular probe set was categorized as 1.5-fold or more up-regulated and if the ratio was ≤ 0.67 then the probe set was categorized as down-regulated by 1.5-fold or more. 141 genes showed 1.5-fold or more up-regulation and 59 genes were down-regulated by 1.5-fold or more in the *Rk*-infected compared with the *Rk*-non-infected treatment (Fig. 3a and 3b). In the compatible interaction 1,060 genes passed the statistical filter (~10% of total expressed probe sets). Among these 1,060 genes 218 were 1.5-fold or more up-regulated and 41 genes were 1.5-fold or more down-regulated in the infected null-*Rk *compared to the non-infected null-*Rk *treatment (Fig. 3a and 3b). In this context it can be noted that in the current study the number of differentially expressed genes is lower than some of the other microarray studies previously published in the field of plant-microbe interactions. One of the reasons may be due to the use of a heterologous GeneChip. Nevertheless, the information generated will be very valuable as this is the first report on the cowpea root-knot nematode interaction. In the final comparison between the two near-isogenic lines infected with *Rk*-avirulent root-knot nematodes, 638 genes (~6% of total expressed probe sets) passed the statistical filter. Among the differentially expressed genes only 20 genes were 1.5-fold or more up-regulated in the infected *Rk *than in the infected null-*Rk *treatment and 100 genes were 1.5-fold or more down-regulated in the infected *Rk *than in the infected null-*Rk *treatment.

Selected genes from all three above comparisons are presented in Tables 1, 2 and 3 with their fold-change ratios and *Medicago truncatula *annotations. The genes were selected based on their fold-change level assuming that the genes with highest fold-change ratio will be likely to have a biological role in the plant-nematode interaction. Lists of all genes passing the 1.5-fold filter are provided in additional files 2, 3 and 4.

**Table 1:** Selected up- and down-regulated genes in infected compared to non-infected resistant cowpea CB46 plants (incompatible interaction) at 9 dpi.*

Probe set name	*Medicago *annotation	E-value	Fold ratio
Genes up-regulated by 4-fold or more			
Gma.4097.1.S1_at	Alcohol dehydrogenase superfamily, zinc-containing	1E-127	11.912
GmaAffx.7738.1.S1_s_at	Homeodomain-related	5E-87	11.64
Gma.17019.1.S1_at	Unknown		10.513
GmaAffx.1441.1.S1_at	Peptidase aspartic	5E-53	10.32
GmaAffx.5726.1.S1_at	Actin/actin-like	1E-68	9.831
Gma.3579.1.S1_at	Serine/threonine protein phosphatase	2E-68	9.656
Gma.11273.1.S1_s_at	Pyruvate decarboxylase	0	9.231
GmaAffx.50860.1.S1_at	Ribosomal protein	2E-42	9.001
Gma.2715.1.S1_at	Concanavalin A-like lectin	0	7.907
GmaAffx.91087.1.S1_s_at	Glycoside transferase	1E-145	7.782
			
GmaAffx.31196.1.S1_s_at	Proteinase inhibitor I9	1E-164	7.52
Gma.876.1.S1_at	Haem peroxidase	1E-100	5.921
Gma.1326.1.S1_a_at	Pectolytic enzyme, Pectin lyase	1E-48	5.814
Gma.8765.1.S1_at	Auxin responsive SAUR protein	1E-72	4.763
GmaAffx.83378.1.S1_at	FAD linked oxidase	1E-104	4.361
GmaAffx.89508.1.A1_s_at	Phenylalanine/histidine ammonia-lyase	1E-107	4.112
Gma.5689.3.S1_s_at	Peptidase, metallopeptidases	3E-63	4.031
			
Genes down-regulated by 3-fold or more			
Gma.3233.1.S1_s_at	Iron superoxide dismutase	1E-95	0.228
Gma.7006.1.S1_at	Expansin 45, endoglucanase-like	1E-119	0.274
Gma.1619.1.S1_at	WD-40 repeat family protein	1E-113	0.303
GmaAffx.80492.1.S1_at	Response regulator receiver	3E-91	0.309
Gma.13643.1.A1_at	Unknown		0.314
Gma.17650.2.S1_at	Hypothetical protein	1E-16	0.319
GmaAffx.89596.1.S1_at	Hypothetical protein	8E-13	0.323

*A p value cut-off of 0.05 was used for t-tests for differential expression.

**Table 2:** Selected up- and down-regulated genes in infected compared to non-infected susceptible null-*Rk *cowpea plants (compatible interaction) at 9 dpi.*

Probe set name	*Medicago *annotation	E-value	Fold ratio
Genes up-regulated by 4-fold or more			
GmaAffx.8712.1.S1_s_at	Haem peroxidase	1E-130	10.98
Gma.17805.1.A1_s_at	Haem peroxidase	7E-47	9.492
Gma.289.1.S1_s_at	Alpha/beta hydrolase	1E-107	8.21
GmaAffx.20156.1.S1_s_at	Glycoside hydrolase	4E-96	6.855
GmaAffx.7738.1.S1_s_at	Homeodomain-related	5E-87	6.594
Gma.4674.1.A1_at	Esterase/lipase/thioesterase	3E-12	4.573
Gma.8525.1.S1_s_at	Haem peroxidase	1E-156	4.351
Gma.2446.1.S1_a_at	Rhodanese-like	5E-08	4.26
GmaAffx.84607.2.S1_at	Phosphate-induced protein 1	2E-19	4.09
			
Genes down-regulated by 2-fold or more			
GmaAffx.89665.1.A1_s_at	Hypothetical protein	4E-64	0.273
GmaAffx.47611.1.S1_s_at	Pollen Ole e 1 allergen	3E-70	0.42
Gma.4750.1.S1_at	Protein of unknown function	4E-56	0.429
GmaAffx.6711.1.S1_at	Auxin Efflux Carrier	8E-61	0.439
Gma.3429.1.S1_at	Dehydrogenase, E1 component	1E-158	0.464
GmaAffx.20418.1.A1_s_at	Similar to unknown protein [*Arabidopsis thaliana*]	1E-19	0.5

*A p value cut-off of 0.05 was used for t-tests for differential expression.

**Table 3:** Selected up- and down-regulated genes in infected resistant (CB46) when compared to infected susceptible (null-*Rk *) cowpea plants at 9 dpi.*

Probe set name	*Medicago *annotation	E-value	Fold ratio
Select up-regulated genes			
GmaAffx.89665.1.A1_s_at	Hypothetical protein	4E-64	2.605
GmaAffx.89786.1.A1_s_at	Hypothetical protein	4E-43	2.343
GmaAffx.76516.1.S1_at	Major facilitator superfamily	2E-21	2.165
GmaAffx.6711.1.S1_at	Auxin Efflux Carrier	8E-61	2.07
GmaAffx.46592.1.S1_s_at	Rhamnogalacturonate lyase	6E-67	1.905
Gma.10150.1.A1_at	2OG-Fe(II) oxygenase	3E-51	1.806
Gma.17019.1.S1_at	Unknown		1.804
Gma.5057.1.S1_a_at	Ubiquinol cytochrome reductase transmembrane region	1E-124	1.798
GmaAffx.42856.1.S1_at	Peptidase S10, serine carboxypeptidase	1E-40	1.693
GmaAffx.31196.1.S1_s_at	Proteinase inhibitor I9	1E-164	1.687
			
Select down-regulated genes			
Gma.289.1.S1_s_at	Alpha/beta hydrolase	3E-75	0.124
Gma.3233.1.S1_s_at	Iron superoxide dismutase	1E-95	0.152
GmaAffx.8712.1.S1_s_at	Peroxidase, putative	1E-130	0.221
Gma.17805.1.A1_s_at	Haem peroxidase	7E-47	0.232
GmaAffx.91763.1.S1_s_at	Xyloglucan endo-transglycosylase	1E-155	0.243
Gma.8525.1.S1_s_at	Haem peroxidase	1E-156	0.279
Gma.2801.1.S1_at	Glycoside hydrolase	1E-124	0.29
Gma.9086.2.S1_at	Cellulose synthase	5E-74	0.3
Gma.1955.4.S1_a_at	Photosystem II oxygen evolving complex protein	3E-13	0.312
Gma.2446.1.S1_a_at	Rhodanese-like	5E-8	0.318

*A p value cut-off of 0.05 was used for t-tests for differential expression.

**Figure 4:**
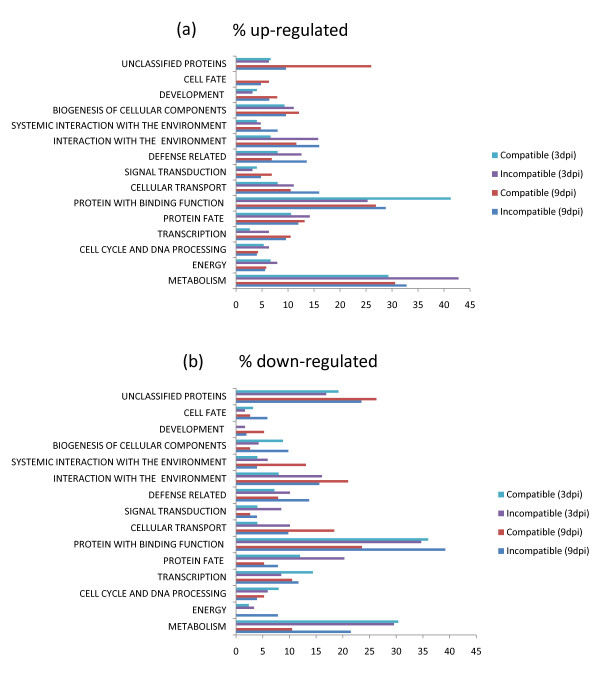
**Functional classification of up- and down-regulated genes in compatible and incompatible cowpea-RKN interactions based on MIPS using homologous sequence of *Arabidopsis***. Only the main functional categories are listed. (a) 1.5-fold or more up-regulated genes in both 9- and 3-dpi samples, (b) 1.5-fold or more down-regulated genes in both 9- and 3-dpi samples.

A gene ontology based analysis was carried out to categorize the differentially expressed genes into different functional classes. In the incompatible interaction the most abundant functional class observed for the up-regulated probe sets (Fig. 4a) was genes involved in metabolism (32.8%), followed by proteins with binding function (28.8%) and genes involved in cell rescue and defense (13.7%). In the compatible interaction the most abundant functional classes in up-regulated probe sets (Fig. 4a) were metabolism (30.6%), proteins with binding function (26.9%), and genes involved in protein fate (13.2%). For down-regulated probe sets the most abundant functional classes in the incompatible interaction (Fig. 4b) were proteins with binding function (39.2%), metabolism (21.5%), and interaction with the environment (15.6%), whereas in the compatible interaction the most abundant classes (Fig. 4b) were proteins with binding function (23.6%), cellular transport (18.4%), and systemic interaction with the environment (13.1%). A significant number of probe sets were also categorized under unclassified or unknown proteins in all the above comparisons.

**Figure 5:**
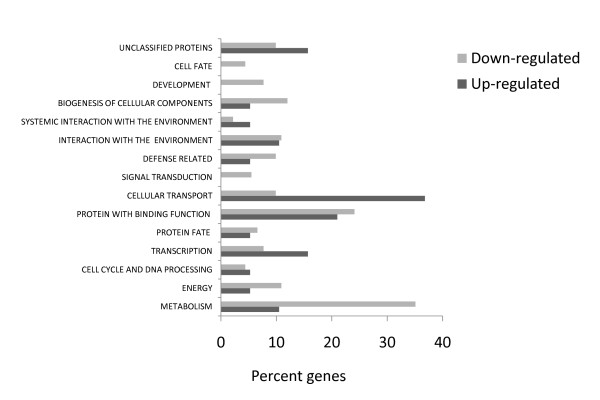
**Functional classification of genes differentially expressed between infected CB46 (resistant) and infected null-*Rk *(susceptible) cowpea roots at 9 dpi, based on MIPS using homologous sequence of *Arabidopsis***. Only the main functional categories are listed.

Genes differentially expressed between the two infected NILs were also functionally classified (Fig. 5). In the probe sets up-regulated in infected resistant CB46 over infected susceptible null-*Rk*, the most abundant classes were cellular transport (36.8%), proteins with binding function (21%), and proteins involved in transcription (15.7%). Among the probe sets which were down-regulated in the resistant genotype over the susceptible genotype, the most abundant functional classes were metabolism (35.1%), proteins with binding function (24.1%), and systemic interaction with the environment (10.9%).

### Gene expression in incompatible and compatible interactions in 3-dpi root samples

Similar to the 9-dpi samples, three different comparisons were made for 3-dpi root samples. In the first comparison gene expression in the resistant *Rk *plants (incompatible interaction) infected with root-knot nematodes was compared with non-infected *Rk *plants. A comparison in gene expression also was made between the infected null-*Rk *(compatible interaction) and non-infected null-*Rk*. Finally, a comparison of gene expression was made between the infected *Rk *and infected null-*Rk *near-isogenic lines.

**Figure 6:**
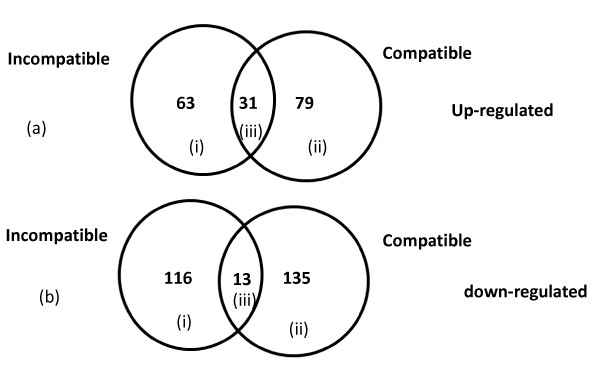
**Venn diagram of differentially expressed genes in incompatible (resistant CB46) and compatible (susceptible null-*Rk*) interactions at 3 dpi**. (a) Genes 1.5-fold or more up-regulated in incompatible (i) and compatible (ii) interactions and genes overlapping between both interactions (iii). (b) Genes 1.5-fold or more down-regulated in incompatible (i) and compatible (ii) interactions and genes overlapping between both interactions (iii).

In the incompatible interaction 746 (~6.9% of total expressed probe sets) genes were significantly differentially expressed based on the statistical test. These genes were then passed through a fold-change filter based on log_2 _ratios. 65 genes showed 1.5-fold or more up-regulation and 129 genes were down-regulated by 1.5-fold or more in the incompatible interaction (Fig. 6a and 6b). In the compatible interaction 623 genes passed the statistical filter (~5.8% of total expressed probe sets). Among these 623 genes 81 were 1.5-fold or more up-regulated and 148 genes were 1.5-fold or more down-regulated in the compatible interaction (Fig. 6a and 6b). In the final comparison between the two near-isogenic lines infected with *Rk*-avirulent root-knot nematodes, 197 genes (~1.8% of total expressed probe sets) passed the statistical filter. Among the differentially expressed genes only 4 genes were 1.5-fold or more up-regulated in the resistant CB46 compared to susceptible null-*Rk*, and 10 genes were 1.5-fold or more down-regulated in the CB46 compared to null-*Rk *plants.

Selected genes from incompatible and compatible interactions are presented in Tables 4 and 5, respectively, with their fold-change ratios and annotations based on the *Medicago *annotation database. Lists of all genes passing the 1.5-fold filter are provided in additional files 5, 6, and 7.

**Table 4:** Selected up- and down-regulated genes in infected compared to non-infected resistant CB46 cowpea plants (incompatible interaction) at 3 dpi.*

Probe set name	*Medicago *annotation	E-value	Fold ratio
Genes up-regulated by 3-fold or more			
Gma.1555.1.S1_a_at	Early light-inducable protein	5E-58	5.126
Gma.18079.1.S1_s_at	Protein kinase	7E-82	4.692
Gma.7289.1.S1_at	Glycosyl transferase	2E-36	4.091
Gma.289.1.S1_s_at	Hydrolase, alpha/beta	3E-75	4.039
GmaAffx.47649.1.S1_at	S-adenosyl-L-methionine:carboxyl methyltransferase	1E-78	3.64
Gma.7224.1.S1_at	C-terminal; Protein kinase	3E-82	3.499
Gma.12211.2.S1_at	Phytochelatin synthetase-like	2E-48	3.463
Gma.8441.1.S1_at	Copper-resistance protein	0	3.263
			
Genes down-regulated by 2.5-fold or more			
GmaAffx.92973.1.S1_s_at	hypothetical protein	3E-15	0.209
GmaAffx.80492.1.S1_at	Response regulator receiver	3E-91	0.22
Gma.2313.2.S1_s_at	Aluminum-induced protein	9E-91	0.273
Gma.10406.1.S1_a_at	Hypothetical protein	2E-81	0.325
GmaAffx.81362.1.S1_at	Endoglucanase-like	7E-60	0.326
GmaAffx.55568.1.S1_at	Histidine kinase related protein	5E-70	0.332
Gma.5992.2.S1_at	Aldehyde dehydrogenase	2E-67	0.334
GmaAffx.57046.1.S1_at	Zinc finger, RING-type	1E-63	0.378
Gma.3712.1.S1_s_at	AKIN gamma - Medicago	0	0.38

*A p value cut-off of 0.05 was used for t-tests for differential expression.

**Table 5:** Selected up- and down-regulated genes in infected compared to non-infected susceptible null-*Rk *cowpea plants (compatible interaction) at 3 dpi.*

Probe set name	*Medicago *annotation	E-value	Fold ratio
Genes up-regulated by 3-fold or more			
Gma.5785.2.S1_at	Glycoside transferase	3E-32	7.464
GmaAffx.84607.2.S1_at	Phosphate-induced protein	2E-19	4.498
Gma.6152.1.S1_at	Multicopper oxidase	2E-98	3.569
GmaAffx.60283.1.S1_at	Zinc finger, RING-type	2E-36	3.416
Gma.15048.2.S1_at	Zinc finger, RanBP2-type	8E-35	3.409
Gma.16367.2.S1_a_at	Ras GTPase	2E-94	3.406
GmaAffx.33748.1.S1_at	Translation factor	1E-76	3.335
GmaAffx.84607.1.S1_at	Phosphate-induced protein 1	3E-24	3.146
			
Genes down-regulated by 3-fold or more			
Gma.10580.2.S1_a_at	PDS1 (phytoene desaturation 1)	1E-106	0.195
Gma.1746.1.S1_s_at	Isocitrate lyase and phosphorylmutase	7E-9	0.265
Gma.2079.3.S1_at	Adenosine/AMP deaminase	1E-158	0.268
Gma.4182.1.S1_s_at	CMP/dCMP deaminase, zinc-binding	9E-89	0.294
Gma.10456.2.S1_a_at	Zinc finger, CCHC-type	1E-161	0.302
GmaAffx.89665.1.A1_s_at	Hypothetical protein	4E-64	0.303
GmaAffx.80492.1.S1_at	Response regulator receiver	3E-91	0.306
GmaAffx.89425.1.A1_s_at	Hypothetical protein	4E-24	0.312

*A p value cut-off of 0.05 was used for t-tests for differential expression.

A gene ontology based analysis was carried out to categorize the differentially expressed genes into different functional classes. In the incompatible interaction the most abundant functional class observed for the up-regulated probe sets (Fig. 4a) was genes involved in metabolism (42.8%), followed by proteins with binding function (25.3%), and genes involved in interaction with the environment (15.8%). In the compatible interaction the most abundant functional classes in up-regulated probe sets (Fig. 4a) were proteins with binding function (41.3%), metabolism (29.3%), and genes involved in protein fate (10.6%). For down-regulated probe sets the most abundant functional classes in the incompatible interaction (Fig. 4b) were proteins with binding function (34.7%), metabolism (29.6%), and genes involved in protein fate (20.3%), whereas in the compatible interaction the most abundant classes (Fig. 4b) were proteins with binding function (36%), metabolism (30.4%), and genes involved in transcription (14.4%). A significant number of probe sets were also categorized under unclassified or unknown proteins in all the above comparisons.

### Comparison of gene expression between 9-dpi and 3-dpi root samples

A comparison of differentially expressed genes was made between the 9-dpi and 3-dpi samples. In the incompatible interaction 188 genes (137 up-regulated by 1.5-fold or more and 51 down-regulated 1.5-fold or more) were uniquely expressed at 9 dpi and 182 (62 up-regulated by 1.5-fold or more and 120 down-regulated 1.5-fold or more) genes were unique for 3 dpi. 12 genes were differentially expressed at both time points (additional file 8). In the compatible interaction 238 genes (198 up-regulated by 1.5-fold or more and 40 down-regulated 1.5-fold or more) were uniquely expressed at 9 dpi and 208 genes (61 up-regulated by 1.5-fold or more and 147 down-regulated 1.5-fold or more) were unique for 3 dpi. 21 genes were differentially expressed at both time points (additional file 9).

## Discussion

Recently a number of interspecies comparisons of gene expression have been carried out including human versus monkeys [26,27], between rodents [28], human versus mouse [29], within *Xenopus *[30], and within *Drosophila *[31]. Cross-species analysis of gene expression in non-model mammals was reported by Nieto-Díaz *et al*. [32]. The reproducibility of probe data obtained from hybridizing deer, Old-World primates, and human RNA samples to the Affymetrix human GeneChip^® ^U133 Plus 2.0 was also compared. The studies showed that cross-species hybridization affected neither the distribution of the hybridization reproducibility among different categories nor the reproducibility values of the individual probes. In plants, the use of heterologous platforms for transcriptome profiling also is becoming more popular. Recently the Affymetrix *Arabidopsis *GeneChip was used to analyze gene expression during seed germination in *Brassica *[33]. The Affymetrix tomato GeneChip was used to survey the early events associated with the potato tuber cold sweetening [34]. The usefulness of the soybean genome array to study cowpea was shown when the Affymetrix soybean GeneChip was used successfully to identify and validate single feature polymorphisms in cowpea [12].

Several microarray studies have been conducted to elucidate the molecular mechanism of the root-knot nematode infection process. Bar-Or *et al*. [15] reported a transcriptome profile of the compatible interaction in susceptible tomato roots infected with root-knot nematodes. Jammes *et al*. [13] and Fuller *et al*. [14] have done similar studies in *Arabidopsis *infected with root-knot nematodes. Several GeneChip microarray studies have also been made recently to investigate the infection process by soybean cyst nematode, *Heterodera glycines *[17,22,20,21].

Microarray studies available so far in the area of plant-nematode interactions mostly examined the compatible or susceptible interaction. There is a dearth of information available for the incompatible plant-root-knot nematode interaction. Very recently Bhattarai *et al*. [16] reported the expression profile of *Mi-1*-mediated incompatible interaction in tomato roots infected with root-knot nematodes. They reported the gene expression profile for only one time point, as early as 24 hours-post inoculation. Incompatible interactions have been monitored for soybean cyst nematode by Klink *et al*. [23,24] for two time points. In our study both compatible and incompatible interactions were studied using the soybean genome array. This is the first insight into the cowpea-root-knot interaction at the transcriptome level. It has already been shown in related work that *Rk*-mediated resistance in cowpea is characterized by a very delayed but strong and effective resistance response [8].

For this study the 9-dpi time point was critical because histologically there are some subtle observable differences between compatible and incompatible interaction at 9 dpi. Though the nematodes were able to maintain normal giant cells in resistant roots at 9 dpi, more vacuolation was evident for the first time in those giant cells when compared to the giant cells in the susceptible roots at the same stage [8]. Among the highly up-regulated genes in the incompatible interaction at 9 dpi was an alcohol dehydrogenase (*Adh*). *Adh *genes are widely known to respond to different biotic stimuli like fungal elicitors [35] and cyst nematodes [20]. This response leads to heavy lignification of cell walls and creates a mechanical barrier for the pathogen. In rice the sequence of 340 kb surrounding the two *Adh *gene loci *Adh1 *and *Adh2 *revealed the presence of 33 putative genes, several of them being resistance gene analogues [36]. Also, among highly up-regulated genes there was a serine threonine protein phosphatase which is known to play a role in negative regulation of defense response in *Arabidopsis *[37]. Also several plant lectins were highly up-regulated in the incompatible interaction at 9 dpi. Plant lectins are carbohydrate binding proteins which are reported to be toxic to several plant pathogens [38]. These findings are consistent with the indications made by Das *et al*. [8] that the vacuoles of giant cells in the resistant genotype might be loaded with certain toxins which leads to developmental and reproductive arrest of the female nematode. Among the down-regulated genes in the incompatible interaction was a superoxide dismutase which generates super oxides [39]. Down-regulation of this gene in the plant prevents reactive oxygen species-mediated cell death which is combined with up-regulation of peroxidases (involved in breakdown of H_2_O_2_) in both incompatible and compatible interactions. These results are consistent with the absence of hypersensitive response (HR)-mediated cell death in the *Rk*-RKN incompatible interaction in cowpea [8]. In the incompatible interaction an expansin was highly down-regulated, and expansins are found to be important for maintaining the specialized feeding structures in hosts by plant parasitic nematodes [23,24]. These findings may indicate that though there is a visible effect of resistance at 9 dpi as indicated by increased vacuolation, the plant is able to generate some defense response against the nematode feeding but the nematode is able to partially suppress the plant defense at this time and continue feeding and development.

When the response of the two near-isogenic lines infected with nematodes was compared at 9 dpi, it was especially noteworthy that a greater number of genes were suppressed in the resistant genotype than were induced or up-regulated compared to the susceptible genotype. This observation is novel because typically in such comparisons more genes are up-regulated than down-regulated in infected resistant plants compared to infected susceptible plants. For example, more genes were induced in the resistant genotype when near-isogenic lines were compared in sugarcane mosaic virus infected maize plants [40] and in wheat plants infected with leaf rust fungus [41]. A plausible explanation for this observation in line with the delayed resistance response is that the defense machinery in the resistant cowpea plant is still suppressed to a large extent and as a result the feeding nematodes are able to maintain functional giant cells even at the 9-dpi stage.

At 3 dpi in both compatible and incompatible interactions, more genes were down-regulated than up-regulated. Jammes *et al*. [13] also reported that there were a significant number of genes down-regulated during giant cell formation in *Arabidopsis *roots, indicating that these suppressed genes might be important negative regulators of nematode parasitism. Though in both incompatible and compatible interactions at 3 dpi there are many induced genes involved in basal defense, only a few genes were common in both interactions. This indicates that despite the similar nature of the infection process in both compatible and incompatible interactions, these two responses have their own molecular signature, unlike *Mi-1 *mediated resistance to RKN in tomato in which many genes are shared between the incompatible and compatible interactions [16]. When expression patterns from both time points were compared we found that there were very few genes which were differentially expressed in both the 3-dpi and 9-dpi samples. The subtle variation in expression of these genes across time points might play a significant role in this biological pathway. For example, an expansin 45 family member (GmaAffx.81362.1.S1_at) was differentially expressed in both 3- and 9-dpi incompatible interactions. At both time points this particular gene was suppressed but the level of suppression was more in the 3-dpi sample when compared to the 9-dpi sample. It is already established that expansin plays a role in feeding site maintenance in both cyst nematode induced syncytia [23,24] and root-knot nematode induced giant cells [16]. Several expansins were found to be up-regulated in syncitia developed by sugarbeet cyst nematode *Heterodera schachtii *in *Arabidopsis *[42]. These subtle variances in gene expression levels might lead to the manifestation of the defense response, and represent areas warranting further investigation through functional analysis.

## Conclusions

In conclusion the results of this study have shown that the typical defense response is still partially suppressed at 9 dpi in resistant cowpea roots. There is an indication that subtle variation of ROS concentration, induction of toxins and other defense related genes play a role in this unique resistance mechanism. It is clear from this study that nematodes are able to keep plant defense responses under considerable control until a high amount of toxins accumulate in the vacuoles which might have resulted from switching on of the plant defense machinery. One of the possible strategies applied by the nematode might be to control the ROS scavenging mechanism in the plants to avoid localised cell death. In *Mi-1*-mediated defense response the nematode is unable to regulate the ROS scavenging and as a result a rapid hypersensitive reaction is triggered upon nematode infection. Further functional analysis of these differentially expressed genes will help us to understand this intriguing plant-nematode interaction in a more precise manner.

## Methods

### Plant material

Two near-isogenic lines (NIL) differing in presence or absence of gene *Rk *were used. The two parents used to develop the NIL were *M. incognita *race 3 resistant cowpea genotype 'CB46' (homozygous resistant, *RkRk*) and a highly susceptible genotype 'Chinese Red' (homozygous susceptible, *rkrk*). The F_1 _was backcrossed to recurrent parent CB46 (BC_1_), homozygous *Rk *plants were discarded in BC_1_F_2, _and non-segregating *rkrk *plants were advanced to the next back-cross (BC_2_). Repeated backcrossing and selection was used to recover the *rkrk *line in the CB46 background. BC_6_F_4 _progenies were used for all the experiments described here. The *rkrk *line is referred to as the null-*Rk *line from here on.

### Nematode inoculum

Eggs of *M. incognita *race 3 (isolate Beltran) cultured on susceptible tomato host plants were extracted from roots using 10% bleach solution [43]. This isolate is avirulent to gene *Rk *in CB46. Eggs were hatched in an incubator at 28°C and J2 were collected in fresh deionized water. The J2 inoculum was prepared according to the experimental requirements.

### Root infections for microarray analysis

Seeds of CB46 and null-*Rk *cowpea lines were surface-sterilized using 10% (v/v) bleach solution and planted singly in seedling growth pouches. Plants were grown under controlled environmental conditions of 26.7°C ± 0.5°C constant temperature and daily light/dark cycles of 16/8 hours. This temperature was used because it lies within the optimum temperature range of 26 - 28°C for development and reproduction of *M. incognita *on cowpea in growth pouches [44]. Each of 100 pouches (50 pouches for each genotype) was inoculated with 3000 J2 in 5 ml of deionized water 12 days after planting (dap). This inoculum level was found to be optimum in a previous study (Das and Roberts, unpublished data) and it generated uniform infection throughout the root system. As a result the amount of infected tissue was maximized. An equal number of pouches were mock-inoculated with 5 ml of deionized water to use as non-infected controls. Infected and non-infected plants were arranged in a completely randomized design. Nematode infected root tissue was excised using a sterile scalpel at 3 days post-inoculation (dpi) and 9 dpi, respectively, under a magnifying glass and flash frozen immediately in liquid nitrogen. In previous studies (Das and Roberts, unpublished) the infected root regions showed swelling at 3 dpi which is indicative of initiation of giant cell formation, and at 9 dpi prominent galling was visible on infected roots. The infected tissue was collected based on these visual indicators. For each biological replicate infected tissue was collected from 7 plants picked randomly and pooled together. This was done in order to obtain enough biological material for RNA isolation. Similarly root tissue was collected from equivalent root regions of the control plants (tissue near root tips of secondary and tertiary roots) and flash frozen. Galled tissue was excised by cutting immediately adjacent to the root-gall in order to minimize the amount of non-infected tissue included in the assays. The harvested tissue was stored at -80°C until RNA isolation. A few infected root pieces were stained in acid fuchsin [45] to confirm the nematode infection.

### RNA isolation

RNA from nematode infected and non-infected root tissue was isolated using RNeasy plant mini kit (QIAGEN Inc., Valencia, CA, USA) according to the manufacturer's protocol. One volume of Plant RNA Isolation Aid (Ambion, Austin, TX, USA) per unit mass of frozen tissue (ml/g) was added before the tissue homogenization step for removal of common contaminants such as polysaccharides and polyphenolics. RNA was treated with RNase-Free DNase set (QIAGEN Inc., Valencia, CA, USA) to digest any genomic DNA which might be present. RNA was quantified using a UV-spectrophotometer. RNA quality and integrity was examined using RNA Lab-On-A-Chip (Caliper Technologies Corp., Mountain View, CA, USA) evaluated on an Agilent Bioanalyzer 2100 (Agilent Technologies, Palo Alto, CA, USA).

### Soybean genome array

Phylogenetic relationships based on the conserved sequences within Papilionoideae legumes imply that *Vigna *(cowpea) is closely related to soybean [46]. Since a commercial cowpea genome array was not available, a soybean genome array (Affymetrix Inc., Santa Clara, CA, USA) was used for transcriptome profiling in cowpea. The soybean genome array contains 37,500 probe sets derived from soybean (*Glycine max *L.) unigenes. This represents 61% of the total probe sets on the chip, with the remainder targeting two pathogens important for soybean research, of which 15,800 (26%) probe sets target *Phytophthora sojae *(a water mold) and 7,500 (12%) probe sets target *Heterodera glycines *(soybean cyst nematode). This array uses probe sets composed of 11 probe pairs to measure the expression of each gene. Each probe pair consists of a perfect match (PM) probe and a mismatch (MM) probe (see also http://www.affymetrix.com/products_services/arrays/specific/soybean.affx).

### Array hybridization

Double-stranded complementary deoxyribonucleic acid (cDNA) was synthesized using SuperScript Double-Stranded cDNA Synthesis Kit (Invitrogen) and T7-oligo (dT) promoter primers. The IVT Labeling Kit (Affymetrix) was then used to synthesize biotin-labeled complementary RNA (cRNA) from template cDNA by in vitro transcription. Twelve to 16 μg labeled cRNA was fragmented by metal-induced hydrolysis to 35-200 base fragments following Affymetrix protocols. 10 μg labeled, fragmented cRNA was then hybridized at 45°C with rotation for 16 h in an Affymetrix microarray Hybridization Oven 320 on Affymetrix soybean genome arrays. The arrays were washed and stained using streptavidin phycoerythrin on an Affymetrix Fluidics Station 450. The arrays were scanned on a Hewlett-Packard GeneArray scanner. cRNA synthesis and array hybridizations were performed in the Genomics Core Facility http://www.genomics.ucr.edu at the University of California, Riverside.

### Data analysis

For 9-dpi samples three biological replicates were used for each of the four treatments (*Rk *infected and non-infected, and *Rk*-null infected and non-infected), requiring 12 soybean GeneChips. For the 3-dpi samples two biological replicates were used for each treatment requiring 8 GeneChips. The data from all 20 chips (CEL and CHP files) are publicly available in Gene Expression Omnibus (http://www.ncbi.nlm.nih.gov/geo/, platform GPL 4592, series GS13631). Expression signals were first analyzed in GeneChip operating software 1.3 (GCOS, Affymetrix Inc.) to determine the "present" probe set list. To detect "present" calls using GCOS software we used all the probe pairs in the probe set as "stat pairs". The definition of the term "stat pairs" is the number of probe pairs per probe set used in the analysis. During data analysis a specified subset of probe pairs can be selected by a probe mask file, but if the default settings are used no probe mask file will be applied and all the probe pairs will be used as "stat pairs" (11 probe pairs in the case of the soybean GeneChip). The detection algorithm uses probe pair intensities to generate a detection p-value and assign a "present", "marginal", or "absent" call. Each probe pair in a probe set has a potential vote in determining whether the measured transcript is or is not "present". The vote is described by the discrimination score (R), which is calculated for each probe pair and compared to a predefined threshold, Tau. Probe pairs with R higher than Tau vote "present" and the voting result is summarized as a p-value. The greater the number of discrimination scores (R) that are above Tau, the smaller the p-value and the more likely the given transcript is truly present in the sample. Only probe sets with a "present" call in all three replicates of at least one treatment were considered to be "expressed".

Data normalization and further analysis was carried out in GeneSpring GX 7.3 (Agilent Technologies, Palo Alto, CA, USA). Robust Multiarray Average (RMA, [47,48]) normalization was performed. Each chip was normalized to the 50th percentile and each gene was normalized to the median. As we were only interested in plant response to nematode infection, all the probe set data from *P. sojae *and *H. glycines *were excluded from any further analysis.

Principal component analysis (PCA) is often used to reduce multidimensional data sets to lower dimensions for summarizing the most important part of the data while simultaneously filtering out the background errors. PCA involves the calculation of the eigenvalue decomposition of a data covariance matrix or singular value decomposition of a data matrix, usually after mean centering the data for each attribute. The results of PCA are usually discussed in terms of component scores and loadings [49]. PCA on conditions (treatments) based on all genes which were present in at least one chip in the 9-dpi and 3-dpi samples were carried out to visualize the overall genome response to nematode infection in the resistant and susceptible cowpea genotypes.

For the 3-dpi samples, with only two biological replicates available, a Pearson correlation coefficient was calculated for normalized values of all probe sets between the two replicates of each treatment to determine the robustness of the data. This analysis was carried out in dChip software [50].

Differentially expressed genes were identified using a one-way analysis of variance (ANOVA) with a p-value cut-off of 0.05. A multiple testing correction was performed using the Bonferroni error correction model [51]. False discovery rate (FDR) was set at 5.0%. Subsequently, differentially expressed genes were filtered for 1.5-fold change in expression level between the control and nematode infected treatment for both genotypes and also between the nematode infected treatments of the resistant and susceptible genotypes.

### Validation of the use of Affymetrix soybean genome array for cowpea transcriptional profiling

In our recent related work [12] the Affymetrix soybean genome array was used successfully to identify single feature polymorphisms in cowpea and the statistical data were validated using PCR amplicon sequencing. Thus, we were able to correctly identify polymorphisms between two cowpea genotypes at a resolution as high as the single nucleotide level. Also, we conducted a small analysis to look at the sequence homology between sequence information files (SIF) of 30 probe sets selected to carry out PCR validation of predicted SFPs and their corresponding cowpea sequences. The homology ranged from 87% to as high as 94.5%. Though this analysis is not exhaustive, it provided a good indication that the homology between cowpea and soybean genomes is quite high at least in the SIF regions from where the probe sets were designed. For this work RNA was used as surrogate for genomic DNA. These data established that the soybean probe sets faithfully measure cowpea transcripts, validating the general reliability of the soybean-based platform for cowpea.

### Annotations and functional classification of genes

The soybean genome array unigene sequences were used to query (using blastx) *Arabidopsis *translated gene models (version 7.0) from The *Arabidopsis *Information Resource (TAIR, http://www.arabidopsis.org) and *Medicago truncatula *2.0 assembly release http://www.medicago.org. Annotations for the Affymetrix soybean probe sets were compiled into a browser called HarvEST:SoyChip which can be accessed online http://www.harvest-web.org or downloaded for Windows installation http://harvest.ucr.edu/. The E value cut-off for the gene annotations was equal to or less than E-10, E0 being a near perfect match.

Gene ontology based classification was obtained by transferring the corresponding *Arabidopsis *gene models to Munich Information Center for Protein Sequences *Arabidopsis thaliana *FunCat database (MIPS, http://mips.gsf.de/proj/funcatDB/search_main_frame.html). *Arabidopsis *gene models were taken from HarvEST:SoyChip.

## Authors' contributions

SD, PAR and TJC designed the experiments. SD performed the research and did the data analysis. SD, PAR and TJC wrote the paper. JDE provided plant materials. All the authors have read and approved the final manuscript.

## Supplementary Material

Additional file 1 **Principal component analysis (PCA) plot of cowpea genome response to nematode infection at 3 days post-inoculation**. Each dot represents the mean of a particular condition (treatment). Click here for file

Additional file 2 **Genes passing 1.5-fold filter in the incompatible cowpea-RKN interaction (9 days post-inoculation)**. Click here for file

Additional file 3 **Genes passing 1.5-fold filter in the compatible cowpea-RKN interaction (9 days post-inoculation)**. Click here for file

Additional file 4 **Genes passing 1.5-fold filter in infected *Rk *compared to the infected null-*Rk *(9 days post-inoculation)**. Click here for file

Additional file 5 **Genes passing 1.5-fold filter in the incompatible cowpea-RKN interaction (3 days post-inoculation)**. Click here for file

Additional file 6 **Genes passing 1.5-fold filter in the compatible cowpea-RKN interaction (3 days post-inoculation)**. Click here for file

Additional file 7 **Genes passing 1.5-fold filter in infected *Rk *compared to the infected null-*Rk *(3 days post-inoculation)**. Click here for file

Additional file 8 **Genes differentially expressed in both 9- and 3-dpi samples in cowpea-RKN incompatible interaction**. Click here for file

Additional file 9 **Genes differentially expressed in both 9- and 3-dpi samples in cowpea-RKN compatible interaction**. Click here for file
