# Supplementary material for: Transcriptional profiling of root-knot nematode induced feeding sites in cowpea (Vigna unguiculata L. Walp.) using a soybean genome array
Source: BMC Genomics. 2010 Aug 19;11:480. doi: 10.1186/1471-2164-11-480 (PMC2996976; doi:10.1186/1471-2164-11-480)
Supplement: Additional file 1 — Principal component analysis (PCA) plot of cowpea genome response to nematode infection at 3 days post-inoculation. Each dot represents the mean of a particular condition (treatment). [file 1471-2164-11-480-S1.PPT]

## Slide 1
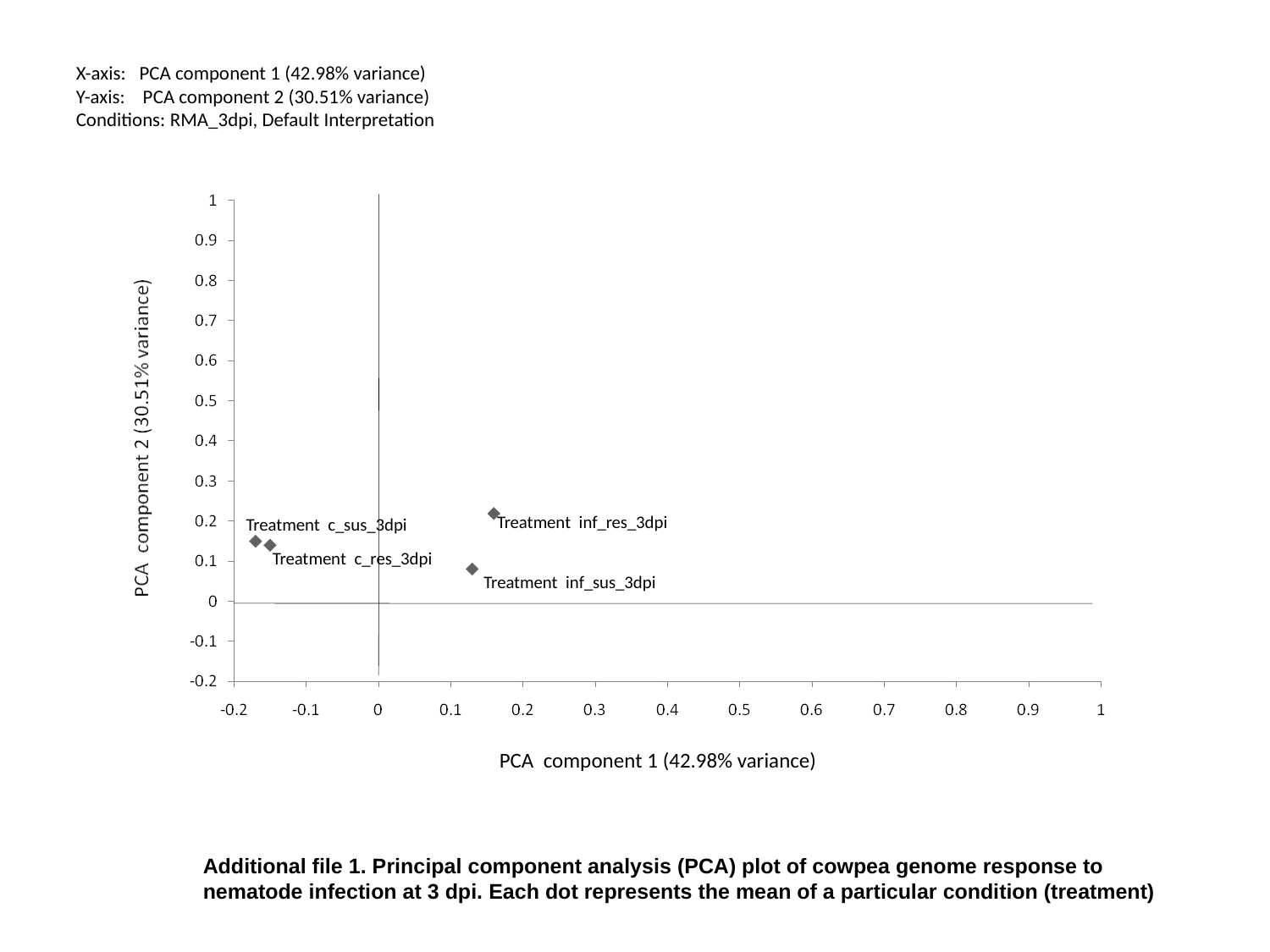

X-axis: PCA component 1 (42.98% variance)
Y-axis: PCA component 2 (30.51% variance)
Conditions: RMA_3dpi, Default Interpretation
Treatment inf_res_3dpi
Treatment c_sus_3dpi
Treatment c_res_3dpi
Treatment inf_sus_3dpi
PCA component 1 (42.98% variance)
Additional file 1. Principal component analysis (PCA) plot of cowpea genome response to nematode infection at 3 dpi. Each dot represents the mean of a particular condition (treatment)
